# Crystal structure of tri­hydrogen bis­{[1,1,1-tris­(2-oxido­ethyl­amino­meth­yl)ethane]­cobalt(III)} trinitrate

**DOI:** 10.1107/S2056989015024664

**Published:** 2015-12-31

**Authors:** Waqas Sethi, Heini V. Johannesen, Thorbjørn J. Morsing, Stergios Piligkos, Høgni Weihe

**Affiliations:** a Department of Chemistry, University of Copenhagen, Universitetsparken 5, DK-2100 Copenhagen Ø, Denmark

**Keywords:** crystal structure, cobalt(III) complex, 1,1,1-tris­(2-hy­droxy­ethyl­amino­meth­yl)ethane, hydrogen-bonding motif, racemic conglomerate

## Abstract

The title compound, [Co_2_(*L*)_2_]^3+^·3NO_3_^−^ [where *L* = CH_3_C(CH_2_NHCH_2_CH_2_OH_1/2_)_3_], has been synthesized from the ligand 1,1,1-tris­(2-hy­droxy­ethyl­amino­meth­yl)ethane. The cobalt(III) dimer has an inter­esting and uncommon O—H⋯O hydrogen-bonding motif with the three bridging hy­droxy H atoms each being equally disordered over two positions. In the dimeric trication, the octa­hedrally coordinated Co^III^ atoms and the capping C atoms lie on a threefold rotation axis. The N atoms of two crystallographically independent nitrate anions also lie on threefold rotation axes. N—H⋯O hydrogen bonding between the complex cations and nitrate anions leads to the formation of a three-dimensional network structure. The compound is a racemic conglomerate of crystals containing either d or l mol­ecules. The crystal used for this study is a d crystal.

## Related literature   

For the crystal structure of the related *cis*-aqua­hydroxido complex of chromium(III), see: Ardon *et al.* (1987 ▸).
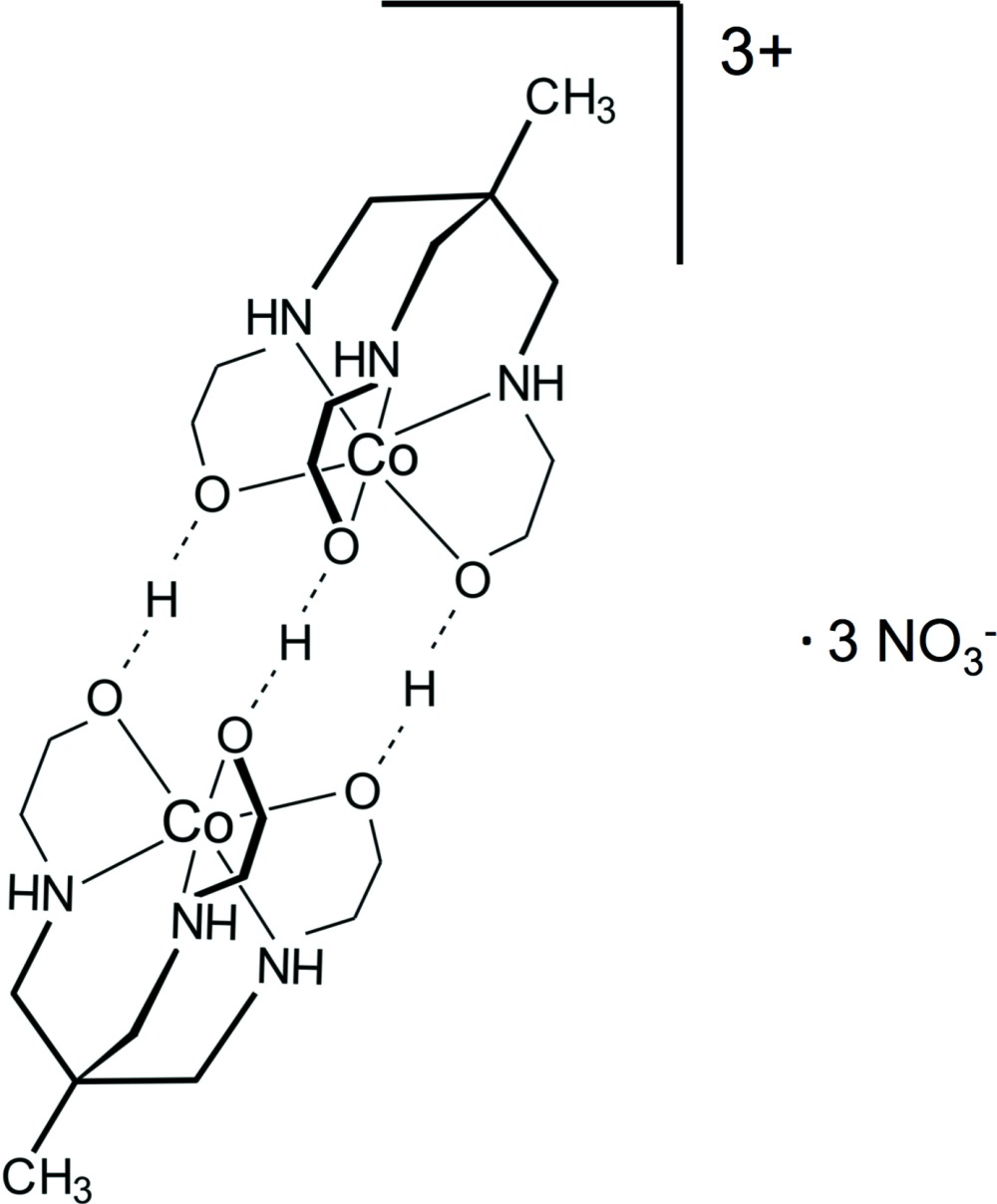


## Experimental   

### Crystal data  

[Co_2_(C_11_H_25.5_N_3_O_3_)_2_](NO_3_)_3_
*M* *_r_* = 799.57
Trigonal, 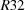

*a* = 8.543 (4) Å
*c* = 39.11 (2) Å
*V* = 2472 (3) Å^3^
*Z* = 3
Mo *K*α radiation
μ = 1.09 mm^−1^
*T* = 122 K
0.31 × 0.25 × 0.15 mm

### Data collection  

Bruker APEXII CCD diffractometer
Absorption correction: multi-scan (*SADABS*; Bruker, 2014 ▸) *T*_min_ = 0.601, *T*_max_ = 0.746
32729 measured reflections
1357 independent reflections
1263 reflections with *I* > 2σ(*I*)
*R*_int_ = 0.074

### Refinement  

*R*[*F* ^2^ > 2σ(*F* ^2^)] = 0.021
*wR*(*F* ^2^) = 0.048
*S* = 0.81
1357 reflections
81 parameters
3 restraints
H atoms treated by a mixture of independent and constrained refinement
Δρ_max_ = 0.24 e Å^−3^
Δρ_min_ = −0.43 e Å^−3^
Absolute structure: Flack *x* determined from 486 quotients [(*I* ^+^)−(*I* ^−^)]/[(*I* ^+^)+(*I* ^−^)] (Parsons & Flack, 2004 ▸)
Absolute structure parameter: 0.011 (8)

### 

Data collection: *APEX2* (Bruker, 2012 ▸); cell refinement: *SAINT* (Bruker, 2012 ▸); data reduction: *SAINT* (Bruker, 2012 ▸); program(s) used to solve structure: *olex2.solve* (Bourhis *et al.*, 2015 ▸); program(s) used to refine structure: *SHELXL2013* (Sheldrick, 2015 ▸); molecular graphics: *OLEX2* (Dolomanov *et al.*, 2009 ▸); software used to prepare material for publication: *OLEX2*.

## Supplementary Material

Crystal structure: contains datablock(s) I. DOI: 10.1107/S2056989015024664/cv5501sup1.cif

**Fig. 1.**
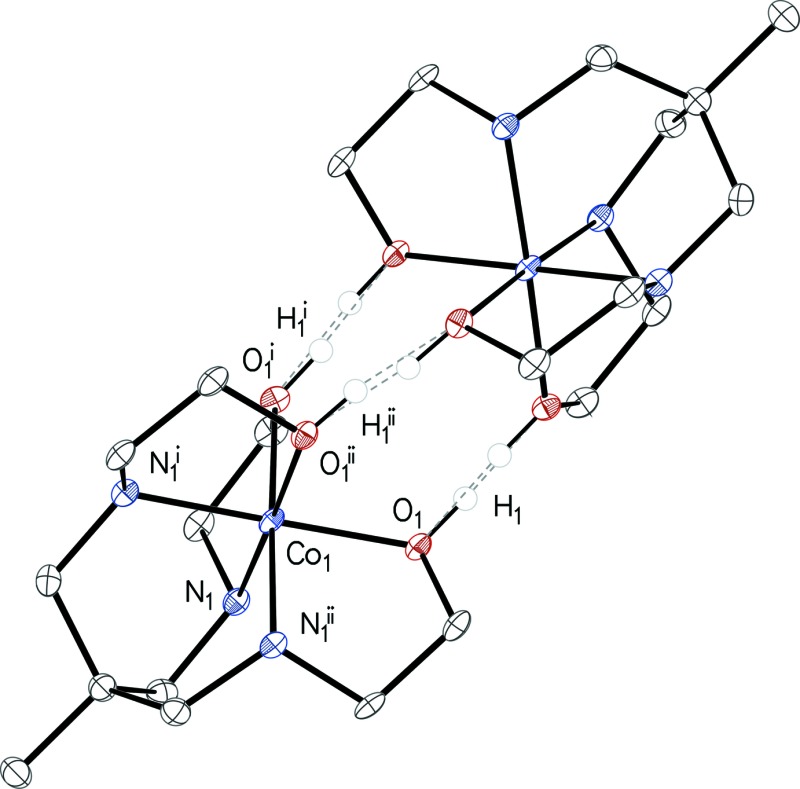
The molecular structure of the dimer cation showing the atomic labels for non-carbon atoms and 50% probability displacement ellipsoids [symmetry codes: (i) −y, x − y, z; (ii) −x + y, −x, z]. H atoms have been removed for clarity, except for the bridging hydroxy H atoms, each of which is disordered over two positions. Dashed lines denote hydrogen bonds.

Structure factors: contains datablock(s) I. DOI: 10.1107/S2056989015024664/cv5501Isup2.hkl

y x y z x y x z . DOI: 10.1107/S2056989015024664/cv5501fig1.tif
The mol­ecular structure of the dimer cation showing the atomic labels for non-carbon atoms and 50% probability displacement ellipsoids [symmetry codes: (i) −*y*, *x* − *y*, *z*; (ii) −*x* + *y*, −*x*, *z*]. H atoms have been removed for clarity, except for the bridging hy­droxy H atoms, each of which is disordered over two positions. Dashed lines denote hydrogen bonds.

CCDC reference: 1443816

Additional supporting information:  crystallographic information; 3D view; checkCIF report

## Figures and Tables

**Table 1:** Hydrogen-bond geometry (Å, °)

*D*—H⋯*A*	*D*—H	H⋯*A*	*D*⋯*A*	*D*—H⋯*A*
O1—H1⋯O1^i^	0.86 (1)	1.59 (1)	2.445 (2)	172 (4)
N1—H1*A*⋯O2	0.98	2.09	3.042 (3)	163

Symmetry code: (i) 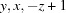.

## supplementary crystallographic information

### S1. Comment

We present here a new hexadentate ligand which, when coordinated to a metal center, facilitates dimer formation through H-bonding (Fig. 1, Table 1). This type of structural motif is rare, but some examples exist in the literature, for example *cis*-aqua-hydroxo complexes of chromium(III) (Ardon *et al.*, 1987).

The ligand, 1,1,1-tris(2-hydroxyethylaminomethyl)ethane, is synthesized from 1,1,1-tris (bromomethyl)ethane and ethanolamine and purified by destillation and column chromatography producing the trihydrochloride. Reaction of a suitable metal salt such as Co(NO_3_)_2_˙6H_2_O and oxidation with hydrogen peroxide affords the title compound (the nitrate salt is much less soluble than the chloride salt).

The title compound, large pink hexagonal single crystals, crystalizes in the trigonal space group *R*32 with the Co—Co axis along the trigonal axis. The compound synthesized is racemic, but upon crystallization it resolves spontaneously to produce a racemic conglamorate. The crystal mounted contains the Δ-form as indicated by a Flack parameter of 0.011 (8) (if the structure is inverted, R1 doubles).

The coordination geometry around the Co ions are close to octahedral with the Co1—N1 distance being 1.95111 (18) Å and the Co1—O1 distance being 1.9314 (14) Å. The O1—Co1—O1 angles are 91.01 (6) ° and the N1—Co1—N1 angles are 92.38 (7) °. The three bridging H-atoms could not be completely located in the Fourier map and were added with a riding model. However, because of the high symmetry and disorder, six equivalent H atom positions exist with the occupancies fixed to 0.5. *ORTEP* plot in Fig. 1 shows all six possible positions. Solving the structure in a space group of lower symmetry does not resolve three H atoms in the trigonal prism formed by the six oxygen atoms, and we take this as a sign that the three H-atoms are disordered over the six positions. The Co—Co distance in the dimer, which is of interrest in regards to future analogues with paramagnetic metal ions, is 4.607 (2) Å. This could give interesting magnetic characteristics for the metal ions like Cr(III) and Mn(III).

### S2. Experimental

Synthesis of the ligand: 1,1,1-tris(bromomethyl)- ethane (60 g, 0,20 mol) was dissolved in ethanolamine (200 ml, 3,3 mol) in a flask fitted with a condenser and a nitrogen in and outlet. After the system was flushed with nitrogen 10–15 min. the mixture was refluxed (170 °C) for 4 h under continued nitrogen flow. The result is a clear pale yellow oil. To remove surplus ethanolamine the reaction mixture was azeotropically distilled using Chlorobenzene (b.p. at approx. 128 °C). After approximately one week ethanolamonium bromide crystallizes and can be removed by filtration. The resulting oil was disolved in 1*M* HCl is put on a cation exchange column (AG 50 W-X2 cation exchange resin (H±form), 5x50cm) and washed with copious amounts of water. The column is then eleuted with 1*M* HCl followed by 2*M* HCl to remove excess ethanolamine. Finally, the column was eluted with 3*M* HCl to isolate the product. The 3*M* HCl eluate was evaporated to dryness to get the product as the hydrochloride salt (an oil). The yield was around 40–50%. 1H NMR (D_2_O): δ = 1.03 (3 H, –CH_3_), 2.94 (6 H, {–CH_2_}_3_), 3.05(6 H, {–CH_2_}_3_), 3.52 (6 H, {–CH_2_}_3_) p.p.m.. C_11_H_24_N_3_O_3_˙3HCl˙10H_2_O (535.9): calcd. C 24.65, N 7.84; found C 24.88, N 7.91.

Synthesis of the title compound: 3 g (10 mmol) Co(NO_3_)_2_˙6H_2_O is dissolved in 10 ml water. 5 g (10 mmol) CH_3_C(CH_2_NHC_2_H_4_OH)_3_˙3HCl˙10H_2_O is dissolved in 10 ml water and added dropwise to the CoII solution. 0.6 g (6 mmol) trimethylamine is also added to the reaction mixture. Concentrated H_2_O_2_ is added dropwise to the reaction mixture until a red powder precipitates. The red powder can be recrystallized from water. Yield: 1.77 g (45%). Co_2_H_45_C_22_N_9_O_15_ (793.518): calcd. C 33.30, N 15.89; found C 33.31, N 15.07.

### S3. Refinement

H atoms were geometrically positioned and refined as riding. The hydroxy atom H1 was placed in the calculated position with occupncy fixed to 1/2, and refined with bond restraint of O—H = 0.87 (1) Å.

### Crystal data

**Table:**

[Co_2_(C_11_H_25.5_N_3_O_3_)_2_](NO_3_)_3_	*D*_x_ = 1.611 Mg m^−^^3^
*M**_r_* = 799.57	Mo *K*α radiation, λ = 0.71073 Å
Trigonal, *R*32	Cell parameters from 6958 reflections
*a* = 8.543 (4) Å	θ = 2.8–28.1°
*c* = 39.11 (2) Å	µ = 1.09 mm^−^^1^
*V* = 2472 (3) Å^3^	*T* = 122 K
*Z* = 3	Prism, pink
*F*(000) = 1260	0.31 × 0.25 × 0.15 mm

### Data collection

**Table:**

Bruker APEXII CCD diffractometer	1263 reflections with *I* > 2σ(*I*)
φ and ω scans	*R*_int_ = 0.074
Absorption correction: multi-scan (*SADABS*; Bruker, 2014)	θ_max_ = 28.1°, θ_min_ = 2.8°
*T*_min_ = 0.601, *T*_max_ = 0.746	*h* = −11→11
32729 measured reflections	*k* = −11→11
1357 independent reflections	*l* = −50→51

### Refinement

**Table:**

Refinement on *F*^2^	Hydrogen site location: mixed
Least-squares matrix: full	H atoms treated by a mixture of independent and constrained refinement
*R*[*F*^2^ > 2σ(*F*^2^)] = 0.021	*w* = 1/[σ^2^(*F*_o_^2^)] where *P* = (*F*_o_^2^ + 2*F*_c_^2^)/3
*wR*(*F*^2^) = 0.048	(Δ/σ)_max_ = 0.003
*S* = 0.81	Δρ_max_ = 0.24 e Å^−^^3^
1357 reflections	Δρ_min_ = −0.43 e Å^−^^3^
81 parameters	Absolute structure: Flack *x* determined from 486 quotients [(*I*^+^)-(*I*^-^)]/[(*I*^+^)+(*I*^-^)] (Parsons & Flack, 2004)
3 restraints	Absolute structure parameter: 0.011 (8)
Primary atom site location: iterative	

### Special details

**Table:**

Experimental. Absorption correction: *SADABS2014*/3 (Bruker, 2014) was used for absorption correction. wR2(int) was 0.1434 before and 0.0855 after correction. The Ratio of minimum to maximum transmission is 0.8053. The λ/2 correction factor is Not present.
Geometry. All e.s.d.'s (except the e.s.d. in the dihedral angle between two l.s. planes) are estimated using the full covariance matrix. The cell e.s.d.'s are taken into account individually in the estimation of e.s.d.'s in distances, angles and torsion angles; correlations between e.s.d.'s in cell parameters are only used when they are defined by crystal symmetry. An approximate (isotropic) treatment of cell e.s.d.'s is used for estimating e.s.d.'s involving l.s. planes.

### Fractional atomic coordinates and isotropic or equivalent isotropic displacement parameters (Å^2^)

**Table:**

	*x*	*y*	*z*	*U*_iso_*/*U*_eq_	Occ. (<1)
Co1	0.0000	0.0000	0.55889 (2)	0.00936 (12)	
O1	0.17219 (17)	0.19761 (18)	0.53088 (3)	0.0118 (3)	
H1	0.176 (5)	0.194 (3)	0.5089 (3)	0.018*	0.5
C5	0.0000	0.0000	0.67637 (7)	0.0177 (7)	
N2	0.6667	0.3333	0.57385 (6)	0.0116 (5)	
O2	0.54318 (19)	0.3734 (2)	0.57427 (4)	0.0237 (4)	
C3	0.1892 (3)	0.0424 (3)	0.62458 (4)	0.0143 (4)	
H3A	0.2763	0.1666	0.6304	0.017*	
H3B	0.2231	−0.0352	0.6367	0.017*	
N1	0.1977 (2)	0.0158 (2)	0.58647 (4)	0.0115 (3)	
H1A	0.3088	0.1208	0.5781	0.014*	
C2	0.2123 (3)	−0.1482 (3)	0.57812 (4)	0.0133 (4)	
H2A	0.1193	−0.2535	0.5900	0.016*	
H2B	0.3295	−0.1298	0.5849	0.016*	
C4	0.0000	0.0000	0.63662 (7)	0.0130 (6)	
C1	0.1742 (4)	0.3620 (2)	0.53957 (4)	0.0144 (4)	
H1B	0.0776	0.3682	0.5278	0.017*	
H1C	0.2885	0.4659	0.5330	0.017*	
N3	0.3333	0.6667	0.6667	0.0389 (11)	
O3	0.3333	0.5219 (4)	0.6667	0.0790 (12)	
H5	−0.123 (2)	−0.032 (3)	0.6841 (4)	0.008 (5)*	

### Atomic displacement parameters (Å^2^)

**Table:**

	*U*^11^	*U*^22^	*U*^33^	*U*^12^	*U*^13^	*U*^23^
Co1	0.00673 (14)	0.00673 (14)	0.01463 (19)	0.00336 (7)	0.000	0.000
O1	0.0113 (7)	0.0081 (7)	0.0151 (6)	0.0042 (6)	0.0011 (5)	0.0002 (5)
C5	0.0201 (11)	0.0201 (11)	0.0129 (14)	0.0100 (6)	0.000	0.000
N2	0.0084 (8)	0.0084 (8)	0.0180 (11)	0.0042 (4)	0.000	0.000
O2	0.0130 (8)	0.0180 (9)	0.0423 (8)	0.0095 (8)	−0.0007 (6)	0.0011 (6)
C3	0.0134 (10)	0.0152 (10)	0.0144 (8)	0.0072 (8)	−0.0034 (7)	−0.0018 (7)
N1	0.0107 (9)	0.0098 (8)	0.0146 (7)	0.0055 (7)	0.0000 (6)	0.0003 (6)
C2	0.0104 (11)	0.0115 (11)	0.0213 (9)	0.0081 (10)	−0.0001 (7)	0.0014 (7)
C4	0.0129 (9)	0.0129 (9)	0.0133 (13)	0.0064 (5)	0.000	0.000
C1	0.0145 (12)	0.0071 (8)	0.0211 (8)	0.0050 (10)	0.0012 (9)	0.0014 (6)
N3	0.0359 (18)	0.0359 (18)	0.045 (3)	0.0179 (9)	0.000	0.000
O3	0.087 (3)	0.0450 (14)	0.119 (3)	0.0435 (14)	−0.060 (2)	−0.0300 (10)

### Geometric parameters (Å, º)

**Table:**

Co1—O1^i^	1.9313 (14)	C3—N1	1.515 (2)
Co1—O1^ii^	1.9313 (14)	C3—C4	1.543 (2)
Co1—O1	1.9314 (15)	N1—H1A	0.9800
Co1—N1	1.9511 (17)	N1—C2	1.503 (3)
Co1—N1^ii^	1.9511 (18)	C2—H2A	0.9700
Co1—N1^i^	1.9511 (17)	C2—H2B	0.9700
O1—H1	0.861 (12)	C2—C1^ii^	1.523 (2)
O1—C1	1.436 (2)	C4—C3^ii^	1.543 (2)
C5—C4	1.555 (4)	C4—C3^i^	1.543 (2)
C5—H5	0.987 (18)	C1—C2^i^	1.523 (2)
N2—O2	1.2612 (16)	C1—H1B	0.9700
N2—O2^iii^	1.2612 (16)	C1—H1C	0.9700
N2—O2^iv^	1.2612 (16)	N3—O3^v^	1.237 (3)
C3—H3A	0.9700	N3—O3	1.237 (3)
C3—H3B	0.9700	N3—O3^vi^	1.237 (3)
			
O1^i^—Co1—O1^ii^	91.01 (6)	Co1—N1—H1A	106.7
O1^i^—Co1—O1	91.01 (6)	C3—N1—Co1	116.71 (12)
O1^ii^—Co1—O1	91.01 (6)	C3—N1—H1A	106.7
O1^i^—Co1—N1^i^	89.92 (7)	C2—N1—Co1	106.72 (11)
O1—Co1—N1^ii^	177.58 (6)	C2—N1—C3	112.72 (14)
O1—Co1—N1^i^	86.74 (7)	C2—N1—H1A	106.7
O1^ii^—Co1—N1^i^	177.58 (7)	N1—C2—H2A	110.5
O1^i^—Co1—N1	177.58 (7)	N1—C2—H2B	110.5
O1^ii^—Co1—N1^ii^	89.92 (7)	N1—C2—C1^ii^	106.35 (14)
O1^ii^—Co1—N1	86.74 (7)	H2A—C2—H2B	108.7
O1—Co1—N1	89.92 (7)	C1^ii^—C2—H2A	110.5
O1^i^—Co1—N1^ii^	86.74 (7)	C1^ii^—C2—H2B	110.5
N1—Co1—N1^ii^	92.37 (7)	C3—C4—C5	107.78 (11)
N1—Co1—N1^i^	92.37 (7)	C3^i^—C4—C5	107.78 (11)
N1^i^—Co1—N1^ii^	92.37 (7)	C3^ii^—C4—C5	107.78 (11)
Co1—O1—H1	124.2 (19)	C3—C4—C3^i^	111.11 (11)
C1—O1—Co1	110.61 (11)	C3^ii^—C4—C3^i^	111.11 (11)
C1—O1—H1	107 (2)	C3^ii^—C4—C3	111.11 (11)
C4—C5—H5	107.8 (10)	O1—C1—C2^i^	107.19 (14)
O2^iii^—N2—O2	119.982 (8)	O1—C1—H1B	110.3
O2^iv^—N2—O2	119.984 (8)	O1—C1—H1C	110.3
O2^iii^—N2—O2^iv^	119.983 (8)	C2^i^—C1—H1B	110.3
H3A—C3—H3B	107.8	C2^i^—C1—H1C	110.3
N1—C3—H3A	109.0	H1B—C1—H1C	108.5
N1—C3—H3B	109.0	O3^v^—N3—O3^vi^	120.000 (3)
N1—C3—C4	112.85 (16)	O3^vi^—N3—O3	119.999 (4)
C4—C3—H3A	109.0	O3^v^—N3—O3	120.001 (9)
C4—C3—H3B	109.0		
			
Co1—O1—C1—C2^i^	36.7 (2)	N1—C3—C4—C3^i^	−71.71 (18)
Co1—N1—C2—C1^ii^	41.38 (17)	N1—C3—C4—C3^ii^	52.55 (19)
C3—N1—C2—C1^ii^	170.77 (17)	C4—C3—N1—Co1	16.05 (19)
N1—C3—C4—C5	170.42 (11)	C4—C3—N1—C2	−108.00 (16)

Symmetry codes: (i) −*y*, *x*−*y*, *z*; (ii) −*x*+*y*, −*x*, *z*; (iii) −*y*+1, *x*−*y*, *z*; (iv) −*x*+*y*+1, −*x*+1, *z*; (v) −*x*+*y*, −*x*+1, *z*; (vi) −*y*+1, *x*−*y*+1, *z*.

### Hydrogen-bond geometry (Å, º)

**Table:**

*D*—H···*A*	*D*—H	H···*A*	*D*···*A*	*D*—H···*A*
O1—H1···O1^vii^	0.86 (1)	1.59 (1)	2.445 (2)	172 (4)
N1—H1*A*···O2	0.98	2.09	3.042 (3)	163

Symmetry code: (vii) *y*, *x*, −*z*+1.
