# Supplementary figures and images for: Crystal structure of tri­hydrogen bis­{[1,1,1-tris­(2-oxido­ethyl­amino­meth­yl)ethane]­cobalt(III)} trinitrate
Source: Acta Crystallogr E Crystallogr Commun. 2015 Dec 31;71(Pt 12):m275–6. doi: 10.1107/S2056989015024664 (PMC4719871; doi:10.1107/S2056989015024664)

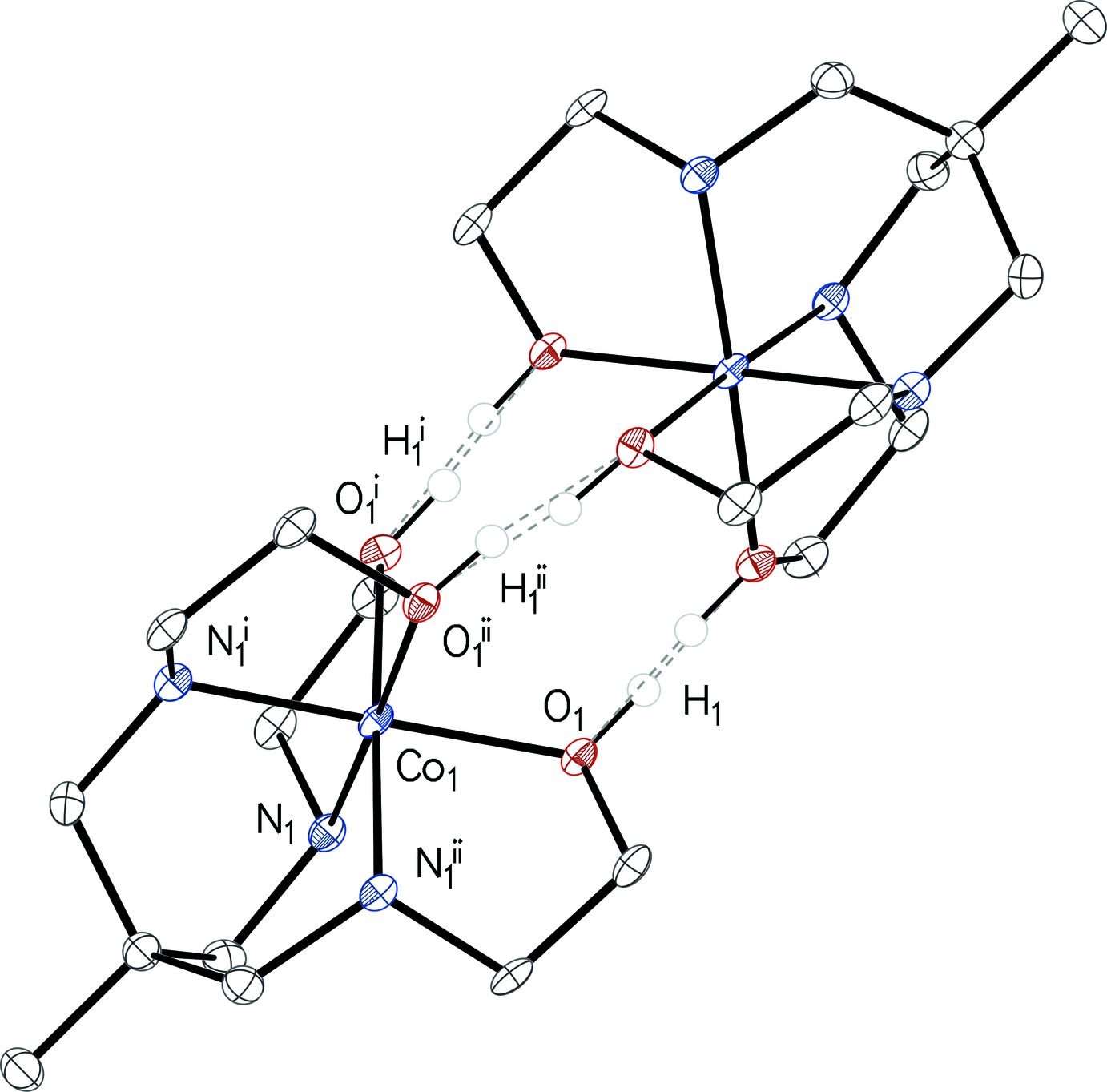

Supplement: Supplementary file 3 [file e-71-0m275-fig1.tif]
